# Pre-stimulus beta power varies as a function of auditory-motor synchronization and temporal predictability

**DOI:** 10.3389/fnins.2023.1128197

**Published:** 2023-03-08

**Authors:** Maren Schmidt-Kassow, Timothy-Niccolo White, Cornelius Abel, Jochen Kaiser

**Affiliations:** ^1^Institute of Medical Psychology, Goethe University Frankfurt, Frankfurt, Germany; ^2^Department of Psychiatry, Psychosomatic Medicine and Psychotherapy, University Hospital, Goethe University Frankfurt, Frankfurt, Germany; ^3^Max Planck Institute for Empirical Aesthetics, Frankfurt, Germany

**Keywords:** anticipation, simultaneous motor activity, predictive timing, self-generated stimulation, temporal expectation, pedaling

## Abstract

**Introduction:**

Auditory-motor interactions can support the preparation for expected sensory input. We investigated the periodic modulation of beta activity in the electroencephalogram to assess the role of active auditory-motor synchronization. Pre-stimulus beta activity (13–30 Hz) has been interpreted as a neural signature of the preparation for expected sensory input.

**Methods:**

In the current study, participants silently counted frequency deviants in sequences of pure tones either during a physically inactive control condition or while pedaling on a cycling ergometer. Tones were presented either rhythmically (at 1 Hz) or arrhythmically with variable intervals. In addition to the pedaling conditions with rhythmic (auditory-motor synchronization, AMS) or arrhythmic stimulation, a self-generated stimulus condition was used in which tones were presented in sync with the participants’ spontaneous pedaling. This condition served to explore whether sensory predictions are driven primarily by the auditory or by the motor system.

**Results:**

Pre-stimulus beta power increased for rhythmic compared to arrhythmic stimulus presentation in both sitting and pedaling conditions but was strongest in the AMS condition. Furthermore, beta power in the AMS condition correlated with motor performance, i.e., the better participants synchronized with the rhythmic stimulus sequence, the higher was pre-stimulus beta power. Additionally, beta power was increased for the self-generated stimulus condition compared with arrhythmic pedaling, but there was no difference between the self-generated and the AMS condition.

**Discussion:**

The current data pattern indicates that pre-stimulus beta power is not limited to neuronal entrainment (i.e., periodic stimulus presentation) but represents a more general correlate of temporal anticipation. Its association with the precision of AMS supports the role of active behavior for auditory predictions.

## Introduction

Behavioral evidence has shown that temporal processing of auditory sequences was facilitated when participants moved in tune with the presented rhythm (Su and Pöppel, 2012). The view that the motor system is involved in sensory processing was further supported by findings that the synchronization of rhythmic movements with target sounds improved attentional selection (Morillon and Baillet, 2017). Furthermore, the motor system appears to be involved in a feedforward/feedback loop, i.e., in preparing motor action based on auditory input regardless of whether or not movements are to be executed (Stephan et al., 2018). Beat perception and predictive timing thus might be controlled by sensorimotor loops.

In this context, beta band activity (approximately 13–30 Hz) plays a crucial role in sensory prediction. Rhythmic tone presentation resulted in a periodic pattern of beta activity in auditory and motor-related cortical regions even in the absence of a motor task (Fujioka et al., 2009, 2012). Beta modulation consisted of two parts: while *beta desynchronization* denotes a beta amplitude reduction following the presentation of an anticipated stimulus, *beta resynchronization* represents an increase of beta amplitude prior to an anticipated stimulus. As beta resynchronization also occurs prior to the omission of an anticipated stimulus (Fujioka et al., 2009, 2012), it seems to reflect an endogenous process and a preparatory mechanism for the auditory and motor systems. Beta resynchronization was more pronounced when participants correctly predicted temporal deviations of the anticipated stimulus, confirming its role in prediction accuracy (Arnal et al., 2015). These findings suggest that beta resynchronization is critically involved in top-down temporal predictions that serve to enhance perceptual processing (Doelling and Poeppel, 2015).

In previous studies, we have shown that auditory-motor synchronization (AMS) affects stimulus encoding (Schmidt-Kassow et al., 2013; Conradi et al., 2016), as reflected by a larger post-stimulus P300 component of the event-related potential in response to rhythmic tones while participants were pedaling compared to sitting still on a stationary bike. This has been interpreted as an indicator of improved encoding and more efficient attention allocation *via* AMS. The current study investigated whether AMS increases beta resynchronization, putatively indicating improved stimulus prediction. We expected to observe such an association for two reasons: first, simultaneous motor activity has been shown to improve temporal prediction (Su and Pöppel, 2012; Morillon and Baillet, 2017). Second, beta-band activity has been associated both with motor functions (Baker, 2007) and top-down control of the sensorimotor system (Engel and Fries, 2010). To take a more specific look at the interaction between motor activity and predictive timing, we compared three types of simultaneous auditory and motor processes by applying a 2-tone oddball paradigm in the following experimental conditions: (1) rhythmic stimulus presentation while participants were pedaling (RP or AMS condition), (2) arrhythmic stimulus presentation while subjects were pedaling (AP), and (3) self-generated stimulus presentation (SP), i.e., participants pedaled at their own pace and stimuli were presented whenever the pedal crossed a light barrier.

Adding an additional self-generated stimulus condition enabled us to compare whether active synchronization with a given stimulus or self-generated stimuli would lead to increased beta synchronization compared to arrhythmic stimulation. If auditory predictions result from outputs from motor regions, one would expect that self-generated stimuli result in better predictions. Higher beta resynchronization preceding self-generated stimulus presentation would indicate that anticipation is primarily driven by the motor system. As participants generated their own (internal) rhythm by actively engaging their motor system, the output from motor regions should help to predict upcoming stimuli. In contrast, higher beta power during the AMS condition would suggest that prediction is primarily driven by the auditory system. Auditory rhythms result in motor synchronization and this in turn facilitates auditory predictions in a circular manner. The better participants synchronize with an auditory signal, the more does the motor system confirm the external rhythm, thereby improving temporal prediction.

We also included two physically inactive control conditions with (4) rhythmic stimulus presentation (RS) or (5) arrhythmic stimulus presentation (AS) while subjects sat still. We did so to replicate the previously reported pre-stimulus beta effect for rhythmic stimuli (Fujioka et al., 2012; Arnal et al., 2015; Doelling and Poeppel, 2015) and to compare the timing effect (rhythmic versus arrhythmic) for the sitting conditions with the pedaling conditions.

Our hypotheses were as follows:

1.) In line with previous work, we expected a timing effect in both settings and across settings, i.e., higher pre-stimulus beta activity for rhythmically versus arrhythmically presented tones. Specifically, this means that we expect positive clusters for the following contrasts: (a) pedaling: rhythmic pedaling versus arrhythmic pedaling (RP > AP), self-generated pedaling versus arrhythmic pedaling (SP > AP) and (b) sitting: Rhythmic sitting versus arrhythmic sitting (RS > AS).
2.) If AMS results in increased predictive timing, we expect higher beta for the rhythmic pedaling versus rhythmic sitting condition (RP > RS), but not for the arrhythmic pedaling versus arrhythmic sitting condition (AP-AS) and a positive correlation between motor performance and pre-stimulus beta power.
3.) If self-generated stimulus presentation results in increased predictive timing, we expect higher beta for the self-initiated pedaling condition compared to the rhythmic sitting condition (SP > RS).
4.) We had no clear hypotheses for the direction of the effect for the contrast self-generated pedaling versus rhythmic pedaling (SP < > RP). As derived above a superiority of the self-generated condition would indicate that auditory predictions result from outputs of motor regions while superiority of the rhythmic pedaling condition would indicate an origin in auditory regions.

## Materials and methods

The experimental design was identical to our previous experiment (Conradi et al., 2016), where we reported post-stimulus event-related potential (P300) data.

### Participants

All thirty participants were undergraduates, Master’s or Ph.D. students at the University of Frankfurt. Four participants had to be excluded because their electroencephalographic (EEG) data was too noisy (see section “Electroencephalographic recording and analysis” below). The remaining 26 participants (15 females, mean age: 21 years, SD = 3.15) did not report any known neurological dysfunction or hearing deficit. Participants received a remuneration of €10,00 per hour.

All methods were carried out according to the guidelines of the Declaration of Helsinki, and the study was approved by the Ethics Committee of the University of Frankfurt Medical Faculty. Participants gave written informed consent to participate in our experiment.

### Experimental design and statistical analyses

The stimulus material consisted of sinusoidal tones of 50 ms duration (sound pressure level 75 dB) generated using MATLAB (The Mathworks, Natick, MA, USA). Standard tones were presented at a frequency of 600 Hz and with a probability of 0.75. Deviant tones, on the other hand, sounded at 660 Hz and were presented with a probability of 0.25. While being presented with the respective tone sequences, the participants were exposed to five different experimental conditions with varying degrees of physical activity. In the rhythmic condition, tones were presented at a constant stimulus onset asynchrony (SOA) of 1,000 ms. In the arrhythmic condition, the SOAs varied randomly between 600 and 1,400 ms (SOAs were evenly distributed around an average of 1,000 ms). To evaluate the effect of simultaneous motor activity on temporal predictability, participants were exposed to both rhythmic and arrhythmic timing conditions, while pedaling on a stationary bike (Conditronic 100 PV/ZR-NS, Dynavit, Kaiserslautern, Germany) at very low intensity (about 50 W), or while sitting still on the bike. Before initiating the rhythmic pedaling condition, participants were informed that they could attempt to synchronize their pedaling rate with the rhythm of tone presentation. Additionally, we also set up a pedaling condition that involved self-generated stimulus presentation. In this condition, participants were initially instructed to pedal at a 1 Hz rate, but then they taught to choose their preferred cycling rate. Consequently, whenever the pedal crossed a light barrier, which was built into the stationary bike (see below), it would trigger the presentation of a new tone. As a result, acoustic stimulation was adapted to the participants’ current pedaling speed. In sum, there were five experimental conditions, i.e., rhythmic stimulation while being inactive (RS), arrhythmic stimulation while being inactive (AS), rhythmic stimulation while pedaling (RP), arrhythmic stimulation while pedaling (AP), and self-generated stimulation while pedaling (SP). Conditions were presented in blocks, i.e., two blocks per condition. Each block contained 148–152 tones and lasted for about 3 min. As a result, each participant finished a total of 10 blocks, whose order was counterbalanced across participants. Oddball sequences were presented by MATLAB (The Mathworks, Natick, MA, USA) running on a Windows PC. Pseudo-randomization ensured that no more than two deviant tones appeared in a row. The tone sequences were presented to the participants using headphones (AKG K271, HARMAN International Industries, Stamford, CT, USA, and Sennheiser MX 365, Sennheiser, Germany). Participants were instructed to silently count the deviant tones while attempting to minimize upper limb movements as much as possible in order to reduce possible EEG artifacts. After each block, participants were asked to report the number of deviant tones they had counted.

All experimental sessions took place in an air-conditioned, windowless, and quiet room. During the stimulation phase, participants faced a white wall to avoid sensory distraction during the experiment.

### Light barrier

For the pedaling conditions, each full rotation was recorded using a customized microcontroller (^®^Arduino),^1^ which featured a light barrier built into the stationary bike. The microcontroller was triggered each time the pedal would cross the light barrier. This experimental setup allowed us to record cycling data during the rhythmic pedaling condition and, during self-generated stimulus presentation, present auditory stimuli at the participants’ favored and individual cadence (Conradi et al., 2016).

### Electroencephalographic recording and analysis

Electroencephalographic activity was recorded using 45 Ag/AgCl electrodes on an elastic electrode cap with 128 equidistant electrodes (Falk Minow Services, Munich, Germany) and Fz as a ground electrode. In order to record vertical electrooculography (vEOG), two electrodes were placed over and under the right eye. Electrodes were referenced on-line to an average reference. Electrode impedances were kept below 20 kΩ. The data were digitized (sampling rate: 1,000 Hz) using a QuickAmp amplifier (Brain Products, Munich, Germany) with an anti-aliasing filter of 250 Hz.

EEG data analysis was performed using MATLAB (The Mathworks, Natick, MA, USA), using the FieldTrip toolbox for EEG/MEG analysis (Oostenveld et al., 2011).

Only epochs of standard tones were considered in the current analysis. In a first step, we segmented the EEG data into epochs of 2,000 ms, which stretched from 1,000 ms prior to stimulus onset to 1,000 ms after stimulus onset. In addition, we applied a band-pass filter (0.1 to150 Hz). Afterward, we performed a three-step procedure in order to automatically remove artifacts: (1) Muscle artifacts were identified using the automatic algorithm, which is integrated into the FieldTrip toolbox. In this context, the data were band-pass filtered (110–140 Hz) and z-transformed. Epochs with a z-score exceeding the threshold value of 20 were discarded. (2) To remove eye artifacts, we performed an independent component analysis (ICA) on the data, using the FastICA algorithm, which is also provided by the FieldTrip toolbox. Components showing correlations with vEOG and exceeding the threshold value of 0.3 were removed from the EEG data using back-projecting all but these components. (3) Epochs showing amplitude differences between two adjacent time points exceeding 20 μV (so-called jump artifacts) were marked as bad trials. If the number of bad trials on a channel exceeded 60%, the channel’s signal was interpolated by the mean of the neighboring channels. Following channel interpolation, subjects with fewer than 100 trials per condition were not included for further analysis (*N* = 4).

After pre-processing and prior to the time-frequency (TF) analysis, the data were resampled to 500 Hz. We followed the protocol for TF analysis by Meijer et al. (2016). Therefore, time-frequency representations (TFRs) for standard tones at each electrode and in each condition were computed using Hanning tapers, using window lengths of 4 cycles per frequency, with 0.25 Hz steps for frequencies between 6 and 36 Hz. We used mirror padding to estimate power values at the beginning and end of each epoch. Single-trial TFRs were baseline-normalized by expressing them as a relative change (in percentage) to the mean of the respective trial [per frequency band (Grandchamp and Delorme, 2011)]. For each experimental condition, we averaged the trials separately, resulting in five TFRs per participant. Grand-average TFRs for each condition were computed by calculating the mean TFR across all subjects.

Beta power differences between relevant conditions were tested using a data-driven, non-parametric and cluster-based permutation procedure (Maris and Oostenveld, 2007) between the following conditions: RS vs. AS and RP vs. AP to assess the timing effect in sitting and pedaling conditions, as well as RP vs. RS, AP vs. AS to assess increased temporal prediction in the AMS pedaling condition. Lastly, we computed contrasts between SP vs. AP/RP and SP vs. RS condition to assess the effect of self-generated stimulation on temporal prediction.

In detail, a dependent-sample *t*-test was conducted to assess beta resynchronization (12 to 30 Hz) differences in a pre-stimulus interval (−600 to 0 ms). To account for multiple comparisons across different time points and electrodes, we performed non-parametric, cluster-based correction method using within-subject permutation (Maris and Oostenveld, 2007). Clusters were defined as adjacent time points and electrodes with uncorrected *p*-values below 0.05. The cluster statistic was calculated as the sum of *t*-values across each cluster. Cluster significance was assessed with a critical alpha level of 0.05 based on the probability distribution of 5,000 permuted data sets.

### Light barrier analysis

In order to examine the participants’ motor performance, the data was analyzed for speed (cadence in Hz) and variability (coefficient of variation) differences between motor conditions (RP, AP, SP) and in particular for AMS performance in the RP condition. AMS performance, that is the ability to adjust one’s pedaling rate to the rhythm of stimulus presentation, was evaluated using inter-beat interval (IBI) deviation (IBD) and calculated for each trial (Leow et al., 2015).


IBI⁢deviation=

$$\frac{\text{mean}\,\left(\left|\mathrm{inter}-\mathrm{pedal}\,\mathrm{interval}\,\mathrm{MINUS}\,\mathrm{inter}-\mathrm{beat}\,\mathrm{interval}\right|\right)}{\mathrm{mean}\,\left(\mathrm{inter}-\mathrm{beat}\,\mathrm{interval}\right)}$$

Inter-beat deviation was calculated by taking the absolute difference between each inter-pedal interval and its corresponding inter-beat interval (always 1 s) and averaging the resulting absolute differences. The average difference is usually divided by the average IBI to normalize to the inter-beat interval and thus control for differences in cue tempo. However, in our paradigm, the IBI was constantly 1s.

We were interested in the relationship between beta power in the pre-stimulus interval and the IBD in the RP condition. Therefore, we correlated the pre-stimulus beta power with the IBD values at electrode sites with highest beta power in the RP-AP condition. The data-driven non-parametric, cluster-based permutation procedure, i.e., randomly permuting the values of the independent variable across subjects, was used to account for multiple comparisons across time points, and electrodes (Maris and Oostenveld, 2007). Clusters were defined as adjacent time points and electrodes with uncorrected *p*-values below 0.05 and cluster statistic was calculated as the sum of *t*-values across each cluster. Cluster significance was assessed with a critical alpha level of 0.05, based on the probability distribution of 5,000 permuted data sets.

For pedaling speed and variability, we computed two repeated-measures ANOVA with the Factor condition (RP, AP, SP) on the dependent variables cadence in Hz and coefficient of variability.

### Code accessibility

All analyses were conducted in MATLAB (MathWorks, Natick, MA, USA). MATLAB scripts for all EEG and motor behavior processing and analysis steps.^2^ These codes were designed for using the open-source toolbox Fieldtrip, version 2019041^3^ and MATLAB 2012b. Data can be made available upon request to the corresponding author (MSK).

## Results

### Behavioral performance

On average, participants made less than one error per condition (SP: 0.68 errors, SD = 0.95; RP: 0.74 errors, SD = 0.93; AP: 1.32 errors, SD = 1.60; RS: 0.79 errors, SD = 0.86; AS: 0.47 errors, SD = 0.51) indicating that they paid attention to the tone sequences. The omnibus ANOVA revealed neither a significant main effect of timing or setting nor an interaction between timing and setting (all *p*-values > 0.09).

### Beta power

**FIGURE 1:**
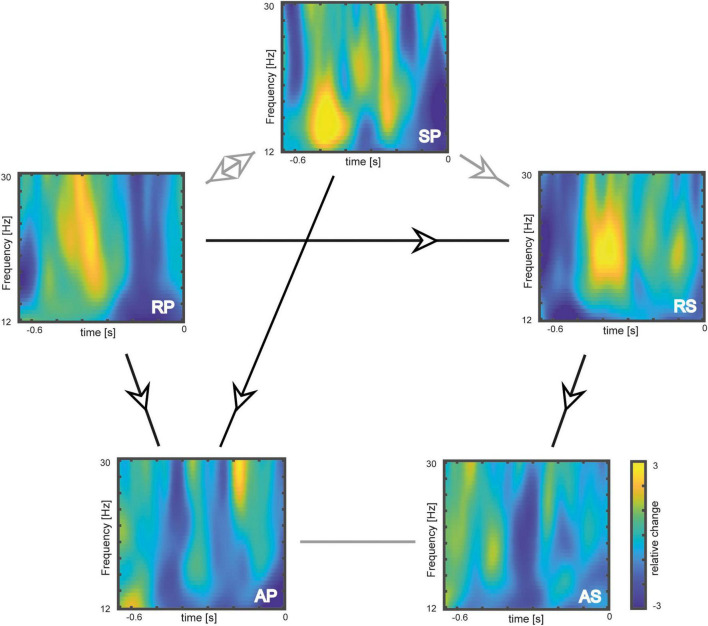
Single experimental conditions: Pre-stimulus beta power grand average across significant electrodes plotted separately for each experimental condition. Beta power is plotted as relative change from the mean in percent. Arrows and lines indicate the hypotheses-guided computed contrasts. Significant effects are plotted in black, non-significant effects are plotted in gray. RP, rhythmic pedaling; AP, arrythmic pedaling; RS, rhythmic sitting; AS, arrhythmic sitting; SP, self-generated pedaling.

**FIGURE 2:**
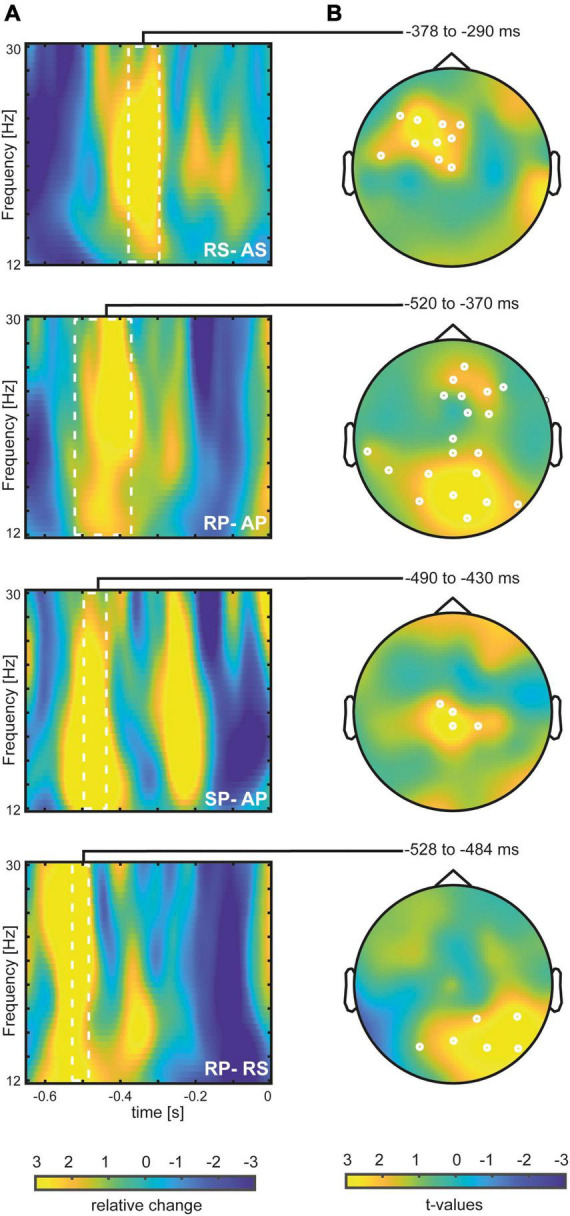
Differences between experimental conditions: Panel **(A)** pre-stimulus beta power grand average across significant electrodes. White rectangles indicate significant time windows. Beta power is plotted as relative change from the mean in percent. Panel **(B)** Topographical distributions of the *t*-values of the pre-stimulus beta power effects. White circles indicate electrode sites that belong to the significant cluster. Upper row: RS-AS condition; second row: RP-AP condition; third row: SP-AP condition; bottom row: RP-RS condition; RP, rhythmic pedaling; AP, arrythmic pedaling; RS, rhythmic sitting; AS, arrhythmic sitting; SP, self-generated pedaling.

First, we tested for pre-tone beta power differences between rhythmic and arrhythmic tone presentation in a pre-stimulus time window ranging from −650 to 0 ms. In all comparisons, we found a stronger pre-stimulus beta power increase in response to temporally predictable tones as compared to arrhythmic stimulation (see Figures 1, 2).

For the comparison of rhythmic versus arrhythmic tones in the sitting condition (RS vs. AS, see Figure 2), we found a positive cluster in a pre-stimulus time window from −374 to −302 ms (*p* = 0.046). For the pedaling condition (RP vs. AP, see Figure 2), we also found a positive cluster in a pre-stimulus time window ranging from −528 ms to −390 ms (*p* = 0.0005). Similarly, the comparison between self-generated pedaling and arrhythmic pedaling (SP vs. AP) resulted in a positive cluster in a time window from −508 to −434 ms (*p* = 0.035).

Most importantly we found a positive cluster for the contrast rhythmic pedaling versus rhythmic sitting (RP vs. RS, −528 to −484 ms, *p* = 0.037) but no cluster for the arrhythmic pedaling versus arrhythmic sitting (AP vs. AS) nor for self-generated pedaling versus rhythmic sitting (SP vs. RS). Furthermore, we found no cluster for the contrast rhythmic pedaling vs. self-generated pedaling. Cluster significance was corrected using the Hochberg procedure (Hochberg, 1988).

### Motor performance

On average, subjects cycled at a cadence of 1.05 Hz (0.046) in the self-generated, 1.02 Hz (0.04) in the rhythmic, and 1.027 Hz (0.068) in the arrhythmic condition. The repeated-measures ANOVA revealed no significant effect of condition (*p* > 0.06).

Concerning motor variability, as measured by the coefficient of variation, participants were descriptively less variable in their motor execution in the rhythmic pedaling (cv = 0.029) and self-generated pedaling (cv = 0.029) condition when compared to the arrhythmic pedaling condition (0.032). However, these differences did not reach significance (*p* > 0.2).

**FIGURE 3:**
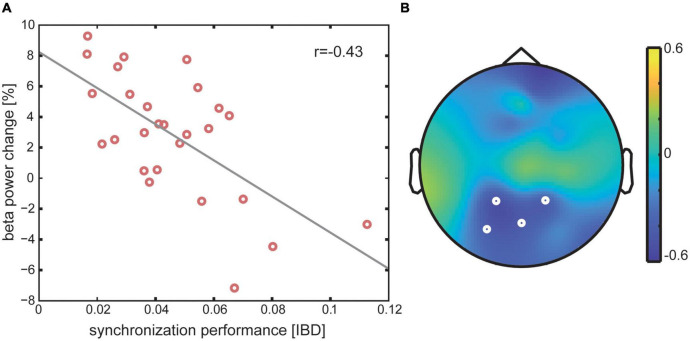
Sensory-motor interaction: Panel **(A)** correlation between pre-stimulus beta power change in percent in the rhythmic pedaling condition and synchronization performance as measured by the IBI deviation. Panel **(B)** topographical distribution of the rho-values of the negative cluster for the respective correlation in the significant time window from –406 to –382 ms. White circles indicate electrode sites that belong to the significant cluster.

However, the IBI deviation (IBD) in the rhythmic pedaling condition [Mean = 0.046 (0.02)] was negatively correlated with pre-stimulus beta frequency power, as revealed by a negative cluster in a time window from −400 to −378 ms (*r* = −0.433; *p* = 0.035, see Figure 3). This means that participants, who deviated more from the presented auditory stimulus rhythm, showed a smaller beta power resynchronization.

## Discussion

The present study investigated whether pre-stimulus beta power varies as a function of auditory-motor synchronization and temporal predictability. Our results show that rhythmic as opposed to random tone presentation leads to an increase of pre-stimulus beta power, both for the sitting and the pedaling conditions. Most importantly this increase is stronger for the AMS compared to the sitting condition and AMS performance is correlated with pre-stimulus beta power, i.e., the better participants synchronize their motor execution, the higher is pre-stimulus beta power in the pedaling condition. This finding indicates that engaging in synchronized motor activity promotes predictive timing processes. A novel aspect of this study was the incorporation of self-generated (SP) tone presentation into a predictive timing paradigm. Although there is a huge body of evidence showing that self-generated stimuli elicit smaller post-stimulus brain responses compared to externally generated sounds (“sensory attenuation,” e.g., Abbasi and Gross, 2020), here we focused on pre-stimulus brain activity and compared three different motor conditions: SP, RP/AMS, and a temporally unrelated motor condition (AP). We provide the novel finding that self-generated tone presentation resulted in a larger enhancement of pre-stimulus beta power compared to arrhythmic tone presentation during physical activity but there was no significant difference between rhythmic motor conditions (SP and RP).

At first glance, our beta effects seem unusually early (300 to 500 ms prior to the onset of the next tone), given that most previous studies have reported predictive beta power peaking immediately preceding the next (expected) tone (Fujioka et al., 2009, 2012). However, this discrepancy might be due to the different experimental setups. Fujioka et al. (2009) asked participants to watch a silent movie while they listened to auditory stimuli, resulting in a pre-attentive experimental condition. In our experiment, participants were shielded from visual distraction and asked to actively direct their attention toward the stimuli by performing a counting task. Furthermore, we used 1,000 ms SOAs, whereas Fujioka et al. (2009, 2012) used much shorter SOAs (390 to 780 ms). In a more recent study, (Fujioka and Ross, 2017) used longer SOAs (400, 800, and 1,200 ms). In the 1,200-ms SOA condition, they found beta power peaks at approximately 400 ms prior to the next beat, which points in a similar direction as our findings. Therefore, the earlier beta peaks in our study may also be attributed to the longer SOA.

In the current study, we have also carefully controlled that our pre-tone beta effect was not simply driven by better aligned periodic movements in the AMS condition (RP) compared to the random pedaling condition (AP) since pre-motor beta modulation might have overridden the auditory-induced beta modulation. We therefore also computed TFRs locked to the motor onset for the rhythmic pedaling and the random pedaling condition and compared both conditions in a pre-motor time window (RPmot vs. APmot, please see Supplementary Figure 1). If our reported pre-stimulus beta effect was motor-driven, we would have expected a comparable beta activity for all motor conditions when time-locked to the motor onset, since neither cadence nor motor variability varied significantly across conditions. However, this was not the case. While, in line with motor performance, RPmot did not differ significantly from APmot when locked to motor onset, the SP condition led to a significantly higher beta peak than RPmot (please see Supplementary material). Here it has to be kept in mind that in the SP condition, motor and tone onset coincided. This means that the beta effect in the SP condition was not only driven by motor activity, but by auditory-driven predictive timing processes. Hence, we argue that the stronger pre-tone beta increases both in the SP and RP conditions compared to the AP condition were driven by a stronger temporal anticipation of the next incoming tone.

Besides the question of whether simultaneous activity enhances temporal predictability, we were particularly interested in disentangling two possibilities of how motor activity may interact with auditory processing: If auditory predictions resulted from outputs from motor regions, we would have expected higher beta power for self-generated stimuli. In contrast, higher beta power for the AMS condition would have suggested that predictions are primarily driven by the auditory system, i.e., auditory rhythms result in motor synchronization and this in turn facilitates auditory predictions in a circular manner.

Our results indicate that both mechanisms result in better predictions compared to random stimulus presentation since we found positive clusters for the contrasts RP vs. AP as well as SP vs. AP. However, interestingly only the AMS condition resulted in a superiority effect compared to the rhythmic sitting condition. Thus, the current results from the RP/AMS condition indicate that active motor synchronization with a given auditory rhythm increases temporal predictions in a circular manner. This finding matches the predictions of the Action Simulation for Auditory Prediction Hypothesis (ASAP, Patel and Iversen, 2014), which assumes that rhythm perception depends on internal predictive models, which help the brain to simulate body movements to entrain neuronal activity in the motor planning system to the beat, as, e.g., in dancing or finger tapping. The main neuronal pathways associated with beat-based timing should involve the dorsal auditory stream, which represents a communication interface between the auditory and motor planning regions. Conversely, information obtained from simulating body movements serves as a predictive signal, which is passed from the motor planning regions to the auditory regions. The ASAP hypothesis serves as a good explanation for our findings regarding the RP condition, as AMS performance was positively associated with beta power, i.e., the better AMS performance, the higher pre-stimulus beta power. In line with the ASAP approach, AMS involves the participant’s active engagement with the environment, which provides bottom-up feedback to the auditory-motor system about the accuracy of the previously generated intrinsic predictions regarding the anticipated auditory input.

Second, we also provided evidence that self-generated tone presentation enhances predictive timing processes compared to random tone presentation while pedaling but not compared to rhythmic tone presentation while sitting. This result complements data from our previous study (Conradi et al., 2016), where we focused on post-stimulus EEG activity, i.e., the P300. In this previous study, we failed to find a beneficial effect of self-generated stimulus presentation compared to random tone presentation while pedaling. This indicates that motor outputs generate auditory predictions (as shown by increased beta) but were not involved in stimulus encoding (as indicated by a missing P300 response). A plausible explanation for this discrepancy was provided by Morillon et al. (2016) who found that temporal predictions as such, and neuronal entrainment to rhythmic stimuli represent separable processes: the enhancement of sensory sensitivity does not depend exclusively on the rhythmic entrainment, but seems to depend more on the participants’ ability to predict when the stimulus will occur (as seen in both SP and RP/AMS conditions). The authors compared three different timing conditions: (a) “periodic predictable,” comparable to our rhythmic condition (RS), and (b) “aperiodic predictable,” where stimuli were presented in streams of decreasing and increasing SOA, i.e., they were not rhythmic, but still predictable. Finally, there was (c) an “aperiodic unpredictable” condition, which corresponds to our arrhythmic condition (AS). They found decreased reaction times to periodic predictable stimuli only, but increased sensory sensitivity for both periodic and aperiodic predictable stimuli (conditions a and b). However, the “aperiodic predictable” condition (b) was also arrhythmic, whereas, in the current study, we compared a rhythmic periodic (or isochronous) with a rhythmic aperiodic (or self-generated, i.e., not isochronous) condition. Our beta power results indicate that temporal predictability in general (i.e., RP/AMS and SP) leads to better preparedness for the incoming stimuli as shown by increased beta power, which is also in line with previous studies (Morillon et al., 2016; Ede et al., 2020; Heynckes et al., 2020), while only rhythmic periodic stimulation is beneficial for the actual evaluation and categorization of the perceived stimulus, as reflected by the previously reported P300 component. However, only AMS leads to a superior timing effect compared with the physically inactive rhythmic condition which is dependent on the participants’ AMS performance. These findings imply that neither motor activity *per se* (as in the arrhythmic pedaling condition) nor the planning of stimulus generation *via* motor activity (as in the self-generated condition) enhances the predictive timing in comparison to rhythmic stimulus presentation. It is the process of actively synchronizing one’s motor response with a rhythmic stimulus presentation that leads to participants’ better preparedness to evaluate the incoming stimulus.

Our pattern of results corroborate the notion that beta oscillations reflect a neuronal network serving as a cortical communication interface between auditory and motor regions (Fujioka et al., 2009; Fujioka and Ross, 2017) and that they play an important role in predictive timing. The degree of pre-stimulus beta power, and hence the accuracy of predictive timing, seems to be correlated positively with the integration of externally generated rhythms into the motor system. As a result, these sensorimotor loops complement each other in order to both facilitate and improve predictive timing and accuracy (Arnal et al., 2015). The importance of these neuronal circuits becomes apparent when brain regions associated with rhythm perception, and/or motor functions are affected by disorders such as Parkinson’s disease (Levy et al., 2002; Hammond et al., 2007; Lei et al., 2019) or stuttering (Falk et al., 2015). In the future, we recommend using different experimental setups with variable types of motor activity (finger tapping, walking, pedaling) and to investigate the functional role of one or the other prediction process and their development across the life span. It would be interesting to know more about which of the two processes (self-generated stimuli or AMS) leads to better temporal predictions in childhood or during aging.

## Data availability statement

The raw data supporting the conclusions of this article will be made available by the authors, without undue reservation.

## Ethics statement

The studies involving human participants were reviewed and approved by the Ethics Committee of the University of Frankfurt Medical Faculty. The participants provided their written informed consent to participate in this study.

## Author contributions

MS-K: conceptualization, methodology, software, data analysis, and writing. T-NW: data collection, data analysis, and writing. CA: conceptualization, methodology, software, and data analysis. JK: writing—reviewing and editing. All authors contributed to the article and approved the submitted version.

## Footnotes

1
www.arduino.cc

2
https://github.com/mschmidt-kassow/prestimbeta

3
https://www.fieldtriptoolbox.org/
