# Supplementary material for: Pre-stimulus beta power varies as a function of auditory-motor synchronization and temporal predictability
Source: Front Neurosci. 2023 Mar 8;17:1128197. doi: 10.3389/fnins.2023.1128197 (PMC10042076; doi:10.3389/fnins.2023.1128197)
Supplement: Supplementary file 1 [file Data_Sheet_1.docx]

Supplementary Material

# Supplementary Data

To control for potential contamination of our anticipation beta effects by motor beta effects, we additionally calculated beta power differences between rhythmic pedalling and arrhythmic pedalling time-locked to the motor onset instead of tone onset (RPmot vs. APmot) as well as between rhythmic pedalling time-locked to the motor onset and self-generated pedalling (i.e. overlapping motor and tone onset; RPmot vs SP). We did so to assess whether our hypothesized pre-stimulus beta increase in the rhythmic pedalling condition could have been due to better aligned periodic movements rather than better anticipation. For our motor control comparisons (RPmot vs APmot, SP vs RPmot) we found a significant positive cluster for the comparison between self-generated pedaling (SP) and rhythmic pedaling locked to the motor onset (RPmot) ranging from -602 to -398 ms (p=. 002). In contrast, we found no beta activity difference between rhythmic and arrhythmic pedaling time-locked to the motor onset (RPmot vs APmot, see Supplementary Figure 1).

*Supplementary Figure 1. Motor control conditions. Row A: Pre-stimulus beta power difference between SP and RPmot and topographical distribution of the t-values of the SP-RPmot effect. The white rectangle indicates the significant time window. White circles indicate electrode sites that belong to the significant cluster. Row B: Pre-stimulus beta power grand average across relevant electrodes plotted separately for RPmot and APmot condition. RPmot=rhythmic pedalling on motor onset, APmot=arrythmic pedalling on motor onset, SP=self-generated pedaling.*
