# Supplementary figures and images for: Pre-stimulus beta power varies as a function of auditory-motor synchronization and temporal predictability
Source: Front Neurosci. 2023 Mar 8;17:1128197. doi: 10.3389/fnins.2023.1128197 (PMC10042076; doi:10.3389/fnins.2023.1128197)

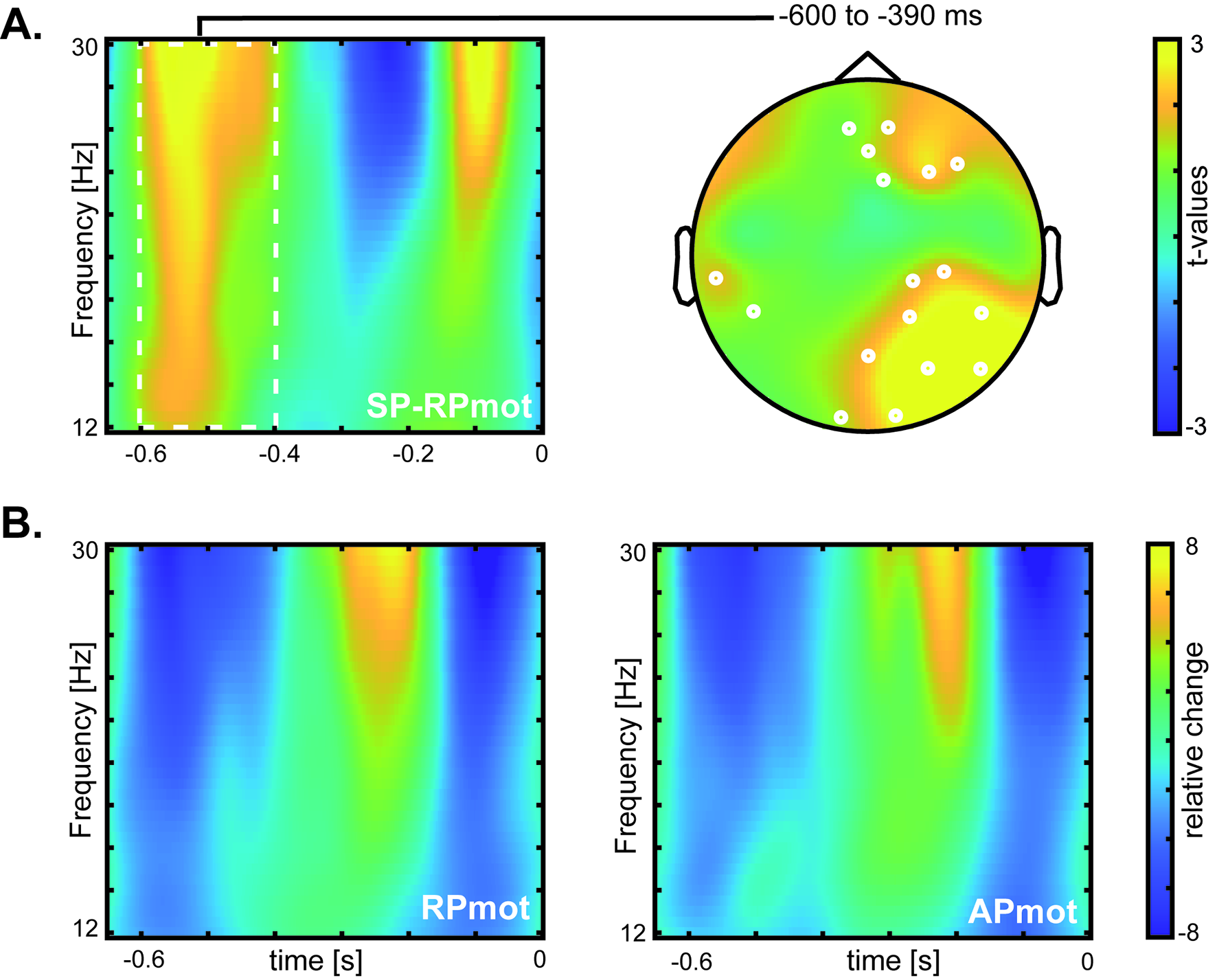

Supplement: Supplementary file 2 [file Image_1.TIF]
